# MDCK-B4GalNT2 cells disclose a α2,3-sialic acid requirement for the 2009 pandemic H1N1 A/California/04/2009 and NA aid entry of A/WSN/33

**DOI:** 10.1080/22221751.2019.1665971

**Published:** 2019-09-27

**Authors:** Ho Him Wong, Kevin Fung, John M. Nicholls

**Affiliations:** a Department of Pathology, University of Hong Kong, Hong Kong; b HKU-Pasteur Research Pole, University of Hong Kong, Hong Kong

**Keywords:** Influenza, receptor, sialic acid, Sda, β-1,4-N-Acetyl-Galactosaminyltransferase 2, B4GalNT2, Madin-Darby Canine Kidney cell, MDCK

## Abstract

Switching of receptor binding preference has been widely considered as one of the necessary mutations for avian influenza viruses, enabling efficient transmissions between human hosts. By stably overexpressing B4GalNT2 gene in MDCK cells, surface α2,3-siallylactose receptors were modified without affecting α2,6-receptor expression. The cell line MDCK-B4GalNT2 was used as a tool to screen for α2,3-receptor requirements in a panel of influenza viruses with previously characterized glycan array data. Infection of viruses with α2,3-receptor binding capability was inhibited in MDCK-B4GalNT2 cells, with the exception of A/WSN/33 (WSN). Infection with the 2009 pandemic H1N1 strains, A/California/04/2009 (Cal04) and A/Hong Kong/415742/2009 (HK09), despite showing α2,6-receptor binding, was also found to be inhibited. Further investigation showed that viral inhibition was due to a reduction in viral entry rate and viral attachment. Recombinant WSN virus with the neuraminidase (NA) gene swapped to A/Puerto Rico/8/1934 (PR8) and Cal04 resulted in a significant viral inhibition in MDCK-B4GalNT2 cells. With oseltamivir, the NA active site was found to be important for the replication results of WSN, but not Cal04.

## Introduction

A switch from α2,3-sialic acid receptor binding to α2,6-sialic acid receptor binding is one of the necessary steps for avian influenza viral adaptation to humans [1,2]. This is in concert with the sialic acid receptor distribution in the upper respiratory tracts of birds and humans [3–5]. While viral haemagglutinin (HA) is generally considered as the main surface glycoprotein for host cell binding and fusion, the co-existing receptor hydrolyzing neuraminidase (NA), has been found to modify receptor binding with a balance between the NA and HA required for efficient replication [6–12].

Ferrets have been used as a model for transmission as they have been considered to have a similar respiratory tract sialic acid distribution and symptoms onset to humans [13,14]. A recent profiling of the ferret respiratory tract using lectins binding and MALDI-TOF however found significant differences [15]. Epithelial cells in the human and ferret upper respiratory tract both bound to the lectins Maackia amurensis agglutinin I (MAA1, α2,3–N glycans) and Sambuccus nigra agglutinin (SNA, α2,6–glycans), with additional binding in the human respiratory tract to Maackia amurensis agglutinin II (MAA2, α2,3–O glycans), however epithelial cells in ferret trachea and lung were also found to possess abundant Sda epitopes (Neu5Ac α2–3(GalNAc β1–4)Gal), that have limited expression in submucus glands of human respiratory tract. Glycan array profiling showed that Sda antigens had low affinity to multiple strains of human, avian and swine influenza viruses, and the lectins MAA1, MAA2, and SNA, but were bound strongly by the Dolichosbiflorus agglutinin (DBA, terminal GalNAc).

**Figure 1.**
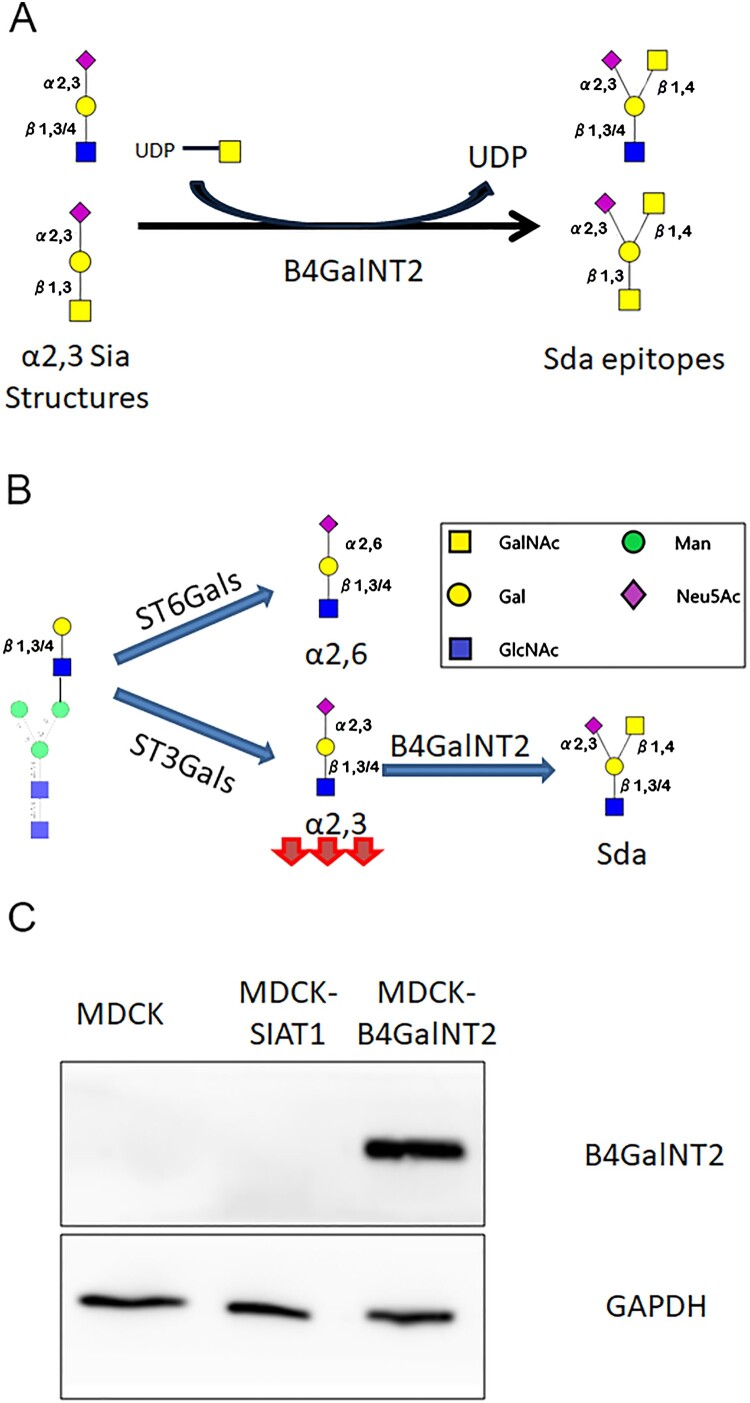
(A) Enzymatic reaction by B4GalNT2. (B) Effect by B4GalNT2 overexpression to convert α2,3 sialic acid receptors into Sda like antigens (C)MDCK, MDCK-SiaT1 and MDCK-B4GalNT2 maintained for 20 passages were lysed for Immunoblot for B4GalNT2 and GAPDH.

B4GalNT2 (β-1,4-N-Acetyl-Galactosaminyltransferase 2) works by transferring a GalNAc in a β1,4-linkage from UDP-GalNAc to the sub-terminal Gal of α2,3-sialyllactosamine and related structures to form Sda (Neu5Ac α2–3(GalNAc β1–4)Gal β1–3/4GlcNAc) and Cad (Neu5Ac α2–3(GalNAc β1–4)Gal β1–3GalNAc) antigens on N-glycans and O-glycans, respectively (Figure 1(A,B)). It cannot catalyze the formation of ganglioside GM2 from GM3 [16]. As a blood group epitope Sda is found abundantly in human saliva, urine and gastrointestinal tract but is reportedly absent in multiple avian species [16–18]. Expression of Sda epitopes protects tissues from being hydrolyzed by exogenous sialidases, and influence microbiota colonization [19–22]. Reduced Sda antigens expression has been linked to colon carcinoma, tumour metastasis and EBV associated gastric cancers [18,23–25]. Pig endothelial cells highly expressing B4GalNT2 gene are known triggers of rejection in pig-to-human xeno-transplantation [26,27].

The relationship of B4GalNT2 and influenza was initially published by Heaton et. al using a CRISPRa screening [28]. We sought to investigate if the MDCK overexpressing B4GalNT2 gene, MDCK-B4GalNT2, could be used to screen for influenza virus with α2,3-sialic acid receptors requirements (Figure 1(B)).

## Materials and methods

### Generation of MDCK-B4GalNT2 by transduction

Human B4GalNT2 cDNA was cloned into pLenti6.3 V5/DEST plasmid (Invitrogen). HEK-293T was transfected with the plasmids to produce pseudo-particles for transduction of MDCK cells. Transduced cells were selected with Blasticidin S (Gibco), and for single clones by limiting dilution. B4GalNT2 expression was confirmed by western blot using a Rabbit anti-B4GalNT2 polyclonal antibody (Abnova, Cat# PAB20841). Expressions of cell surface epitiopes were confirmed by immunocytochemistry (ICC) and enzyme-linked lectin assay (ELLA).

### Immunocytochemistry and binding assay for lectins and antibodies

Cells seeded on glass coverslips were stained with biotinylated SNA (Vector Labs, Cat# B-1305-2), MAA1 (Vector Labs, Cat# B-1315-2), MAA2 (Vector Labs, Cat# B-1265-1), DBA (Vector Labs, Cat# B-1035-5) or mouse monoclonal CT-1 antibody (courtesy of Dr Karl Klisch) and then with alkaline phosphate conjugated streptavidin (Vector Labs). Red colours were developed with Vector Red substrate kit (Vectors Labs), and cell nuclei were counterstained blue with Mayer’s Hematoxylin.

Lectins binding was quantified by enzyme-linked lectin assay (ELLA). Cell lysate was coated on polystyrene microplates at 2 μg/ml. Plates were blocked and then stained with biotinylated MAA1, MAA2, SNA and DBA. HRP conjugated streptavidin (Biolegend) was then added. Signals were developed using TMB solution (Biolegend), and stopped with 2N sulphuric acid before signal saturation. Absorbance at 450 nm was immediately measured.

### Virus culturing and viral infection

WSN, WSN mutants and Cal04 were generated by reverse genetics as described previously, and was used in the first passage [29]. Cal04 NA, Cal04 HANA and PR8 NA were WSN mutants with the specified coat protein being replaced. PR8 and its PB2 mutants were a gift from Professor LLM Poon, University of Hong Kong [30]. The remaining influenza viruses were selected according to previous published glycan array data from our group [4], or from Consortium for Functional Glycomics (CFG, http://www.functionalglycomics.org/). All above viruses were used within passage 5 and propagated in MDCK cells with supplementation of TPCK-Trypsin (Sigma-Aldrich), except for A/Duck/Bavaria/1/1977, which was egg propagated.

For viral infection experiments, MDCK and MDCK-B4GalNT2 cells were incubated with influenza viruses at the multiplicity of infection (MOI) specified for 30 min at 37°C, before replacing with new infection medium. Cell supernatants were collected at times specified for viral titre quantification with standard plaque assay method.

### FACS analysis of infected cells

Cells infected with influenza virus at MOI 1 for 30 min were further incubated for 8 h after changing medium. Trypsin/EDTA detached cells were fixed with 4% formaldehyde and permeabilized with 0.1% Triton X-100 before staining with fluorescein isothiocyanate (FITC)-conjugated anti-influenza A NP antibody (ARGENE, Ref: 12-030). FITC positive cells were acquired by a BD FACS Calibur and analyzed with FlowJo (FlowJo, LLC).

### Virus labelling and attachment assay

Viruses were labelled with Octadecyl rhodamine B (R18) as described previously [31,32]. Labelled viruses were added to trypsin detached cells in ice-cold PBS and allowed for attachment at 4°C for 60 min. Cells were washed with ice-cold PBS and fixed with 4% formaldehyde before acquiring by a BD FACS Aria Special Order Research Product. Results were analyzed by Flowjo (FlowJo, LLC).

### Modified plaque assay

For assay with oseltamivir, influenza viruses diluted to around 60 PFU/mL were added into each well seeded with MDCK or MDCK-B4GalNT2 cells. Cells were incubated at 37°C for 60 min before rinsing the wells with PBS, and overlaid with 1% DMEM agarose containing 1ug/mL TPCK-Trypsin. 10nM oseltamivir carboxylate was added only during viral dilution and infection for co-treated group, only in the 1% agarose/DMEM after incubation for the post-treat group or not added in any step for untreated group.

For assessment of the rate of viral entry, influenza viruses diluted to around 60 PFU/mL (at 60 min) were added into wells seeded with MDCK or MDCK-B4GalNT2 cells. Cells were incubated at 37°C for times specified, before rinsing the wells with PBS and overlaid with 1% agarose/DMEM containing 1ug/mL TPCK-Trypsin.

For both assays, plates were incubated at 37°C for 72 h before formaldehyde fixation and staining with crystal violet.

### Neuraminidase treatment of cells

MDCK and MDCK-B4GalNT2 cells were incubated in DMEM with or without Vibrio Sialidase (SiaV) (Roche) at a final concentration of 100 mU. Cells were incubated for 2 h at 37°C before changing medium, and infection with virus.

## Results

### Transduction of B4GalNT2 to MDCK

A single clone of B4GalNT2 transduced MDCK with the highest protein expression was selected for further experiments. Immunoblot after 20 passages confirmed B4GalNT2 protein remained stably overexpressed (Figure 1(C)).

### Characterization of glycan profile

**Figure 2.**
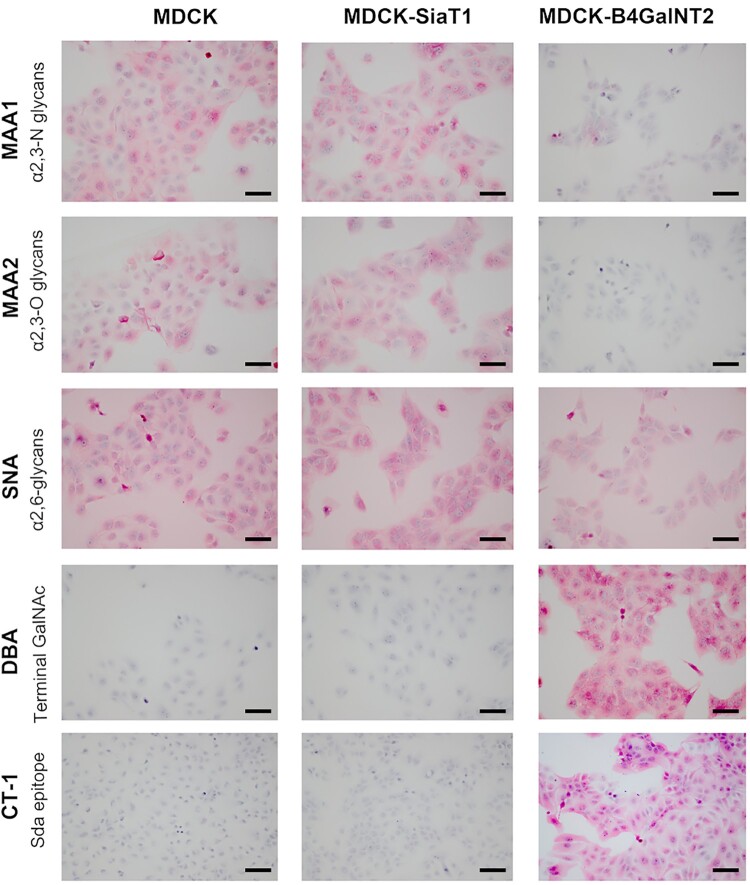
Immunocytochemistry of MDCK, MDCK-SiaT1 and MDCK-B4GalNT2 cells with lectins MAA1, MAA2,SNA, and DBA and monoclonal antibody CT-1. Scale bar = 100 μm.

To ensure B4GalNT2 overexpression was functioning to convert α2,3-sailic acid receptors into Sda-like epitopes, cells were stained with MAA1, MAA2, SNA, DBA and CT-1 antibody (Sda epitopes) by ICC (Figure 2). In contrast to MDCK and MDCK-SiaT1, MDCK-B4GalNT2 showed negative binding to MAA1 and MAA2, but strongly bound to DBA and CT-1 antibody. SNA bindings were similar in MDCK and MDCK-B4GalNT2, but slightly stronger in MDCK-SiaT1.

**Figure 3.**
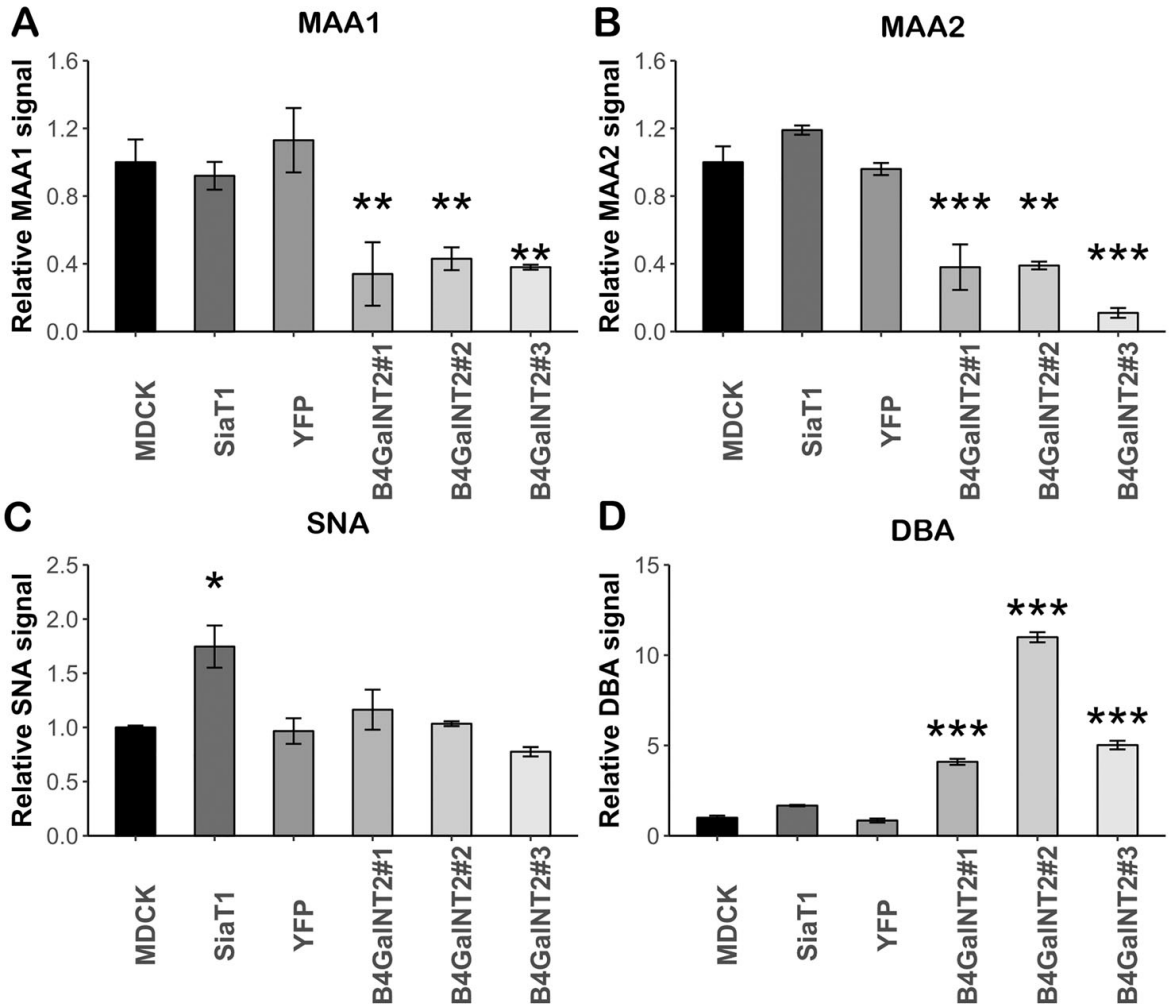
Enzyme-linked lectin assay of cell lysates from MDCK, MDCK-SiaT1 (SiaT1), MDCK-YFP (YFP) and 3 MDCK-B4GalNT2 clones bound by (A)MAA1, (B) MAA2, (C) SNA_and (D) DBA. Signals are normalized to wild-type MDCK cells. Mean ± 1sd, *n* = 3. *p < 0.05, **p < 0.01, ***p < 0.001, one way-ANOVA.

ELLA was performed to numerically measure lectin binding levels. 2 more clones of B4GalNT2 transduced MDCK (MDCK-B4GalNT2 #2 and #3) and a polyclonal YFP transduced MDCK cell line (MDCK-YFP) were used to ensure the changes in surface glycan expressions were the specific effect of transduced B4GalNT2 gene. In keeping with ICC staining, MDCK-B4GalNT2 showed a significant reduction in MAA1 and MAA2 bindings (Figure 3(A,B)). In keeping with the results from Matrosovich et al, SNA binding (Figure 3(C)) of MDCK-SiaT1 was nearly double of that of MDCK and MDCK-B4GalNT2 [33]. There was a significant increased DBA binding in MDCK-B4GalNT2 (Figure 3(D)).

In conclusion, upon overexpression of B4GalNT2 gene, MDCK-B4GalNT2 expresses an undetectable level of α2,3-sialic acid receptors on O-glycans and N-glycans but a high level of Sda epitopes without significantly changing α2,6-sialic acid receptor content.

### Infection with PR8 and its PB2 mutants

Because of the dramatic depletion in bindings of MAA1 and MAA2, we hypothesized that infection by influenza viruses with α2,3-binding capabilities were to be inhibited in MDCK-B4GalNT2.

**Figure 4.**
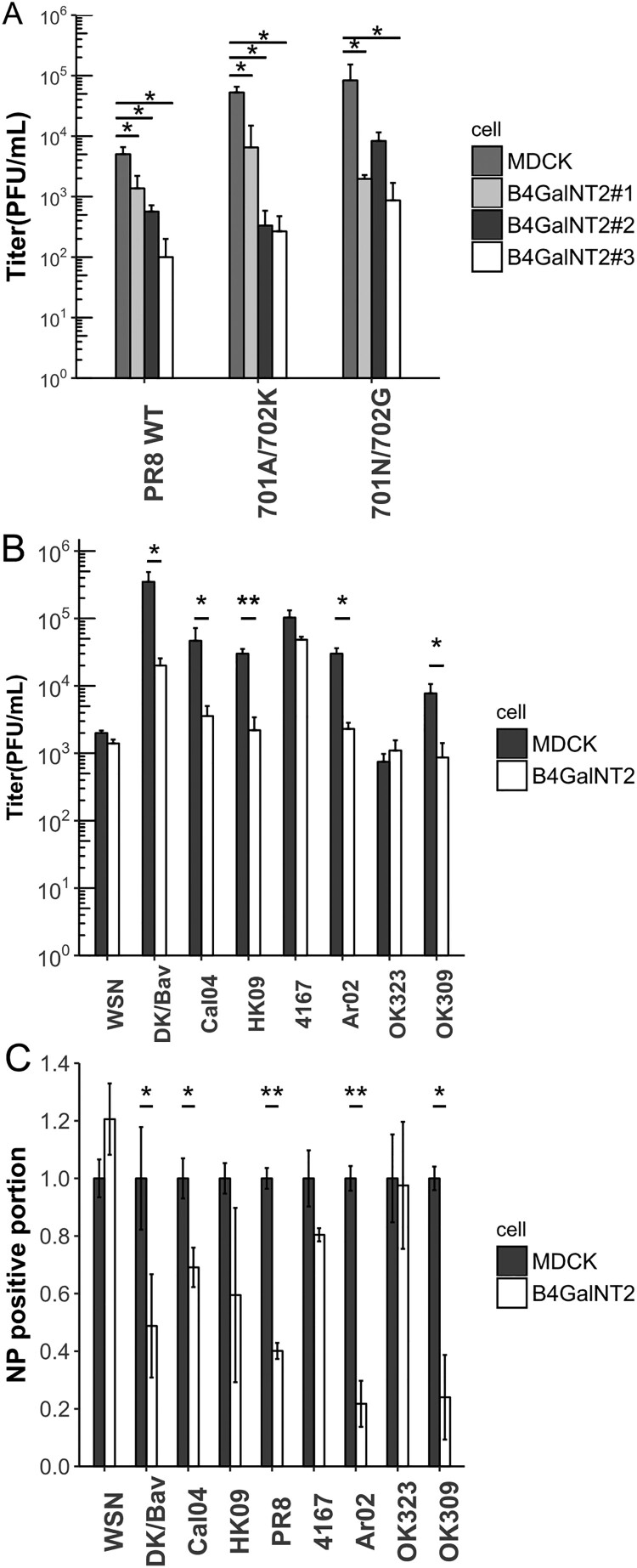
(A) MDCK and MDCK-B4GalNT2 were infected with PR8 and its PB2 mutants at MOI 1 for 8 h. Mean ± sd, *n* = 3. (B) MDCK and MDCK-B4GalNT2were infected with WSN, Dk/Bav, Cal04, HK09, 4167, OK323, Ar02 and OK309 at MOI 4 for 8 h. Mean ± 1sd, *n* = 3. (C) Proportion of NP positive cells normalized to that of wild type MDCK. Mean ± sd, *n* = 2. *p < 0.05, **p < 0.01, ***p < 0.001, 2 tailed T-test.

Multiple clones of MDCK-B4GalNT2 were infected with wild type PR8, a dual binding virus (Table 1) and its PB2 mutants, 701A/702 K and 701N/702G, with altered polymerase activities at MOI 1 for 8 h (Figure 4(A)) [30]. Wild-type PR8 titres were 10 folds lower in MDCK-B4GalNT2 clones. Despite PB2 mutants showing 1 log higher titre than wild type PR8, titre differences between MDCK and MDCK-B4GalNT2 clones remained. The results evidenced that B4GalNT2 overexpression inhibited PR8 infection with different polymerase activities.

**Table 1.** List of the 9 strains of influenza A viruses used in the experiments. Abbreviations, subtypes, sialic acid receptor binding preferences and species of origin are shown.

Strain	Abbreviation	Subtype	Binding preference	Origin	Reference
A/WSN/33	WSN	H1N1	α2,3, α2,6	Human	CFG primscreen_2664, 2933, 3720, 5336 [11,34,35]
A/Duck/Bavaria/1/1977	DK/Bav	H1N1	α2,3	Duck	[4]
A/Puerto Rico/8/1934	PR8	H1N1	α2,3, α2,6	Human	CFG primscreen_1367, 2055
A/California/04/2009	Cal04	H1N1	α2,6	Human	CFG primscreen_2777, 3103, 3104, 3128
A/Hong Kong/415742/2009	HK09	H1N1	α2,6	Human	[4]
A/Swine/Arkansas/2976/2002	Ark02	H1N2	α2,3, α2,6	Swine	[4]
A/Swine/Hong Kong/4167/1999	4167	H1N1	α2,6	Swine	[4]
A/Oklahoma/309/2006	OK309	H3N2	α2,3, α2,6	Human	[4,6]
A/Oklahoma/323/2003	OK323	H3N2	α2,6	Human	[4,6]

### Testing influenza strains with glycan array data

A list of 8 more strains of influenza virus from different origins and subtypes were selected based on available glycan array data (summarized in Table 1). MDCK and MDCK-B4GalNT2 were infected at MOI 4 for 8 h to investigate viral single cycle growth kinetics (Figure 4(B)). For α2,3-binding viruses, viral titres of DK/Bav dropped more than 20 folds in MDCK-B4GalNT2. Despite showing α2,3-binding ability in previous reports, replication of WSN was not affected. For α2,6-binding viruses, OK323 and 4167 showed no significant reduction in viral titres, but Cal04 and HK09 showed a 10-fold reductions of viral titres in MDCK-B4GalNT2 cells. For dual binding viruses, titres of Ar02 and OK309 reduced by 1 log in MDCK-B4GalNT2.

Infected cells were stained for NP and analyzed with FACS for the proportion of cells infected (Figure 4(C)). Results from FACS were in coherent with that of viral titres that smaller proportions of cells were NP positive in MDCK-B4GalNT2 infected with strains that single round growth were inhibited by B4GalNT2 overexpression. The results clearly showed that the reductions in resultant viral titres in DK/Bav, Cal04, HK09, PR8, Ar02 and OK309 infected MDCK-B4GalNT2 were due to reductions in viral infected cells.

### Overexpression of B4GalNT2 reduced rate of viral entry

**Figure 5.**
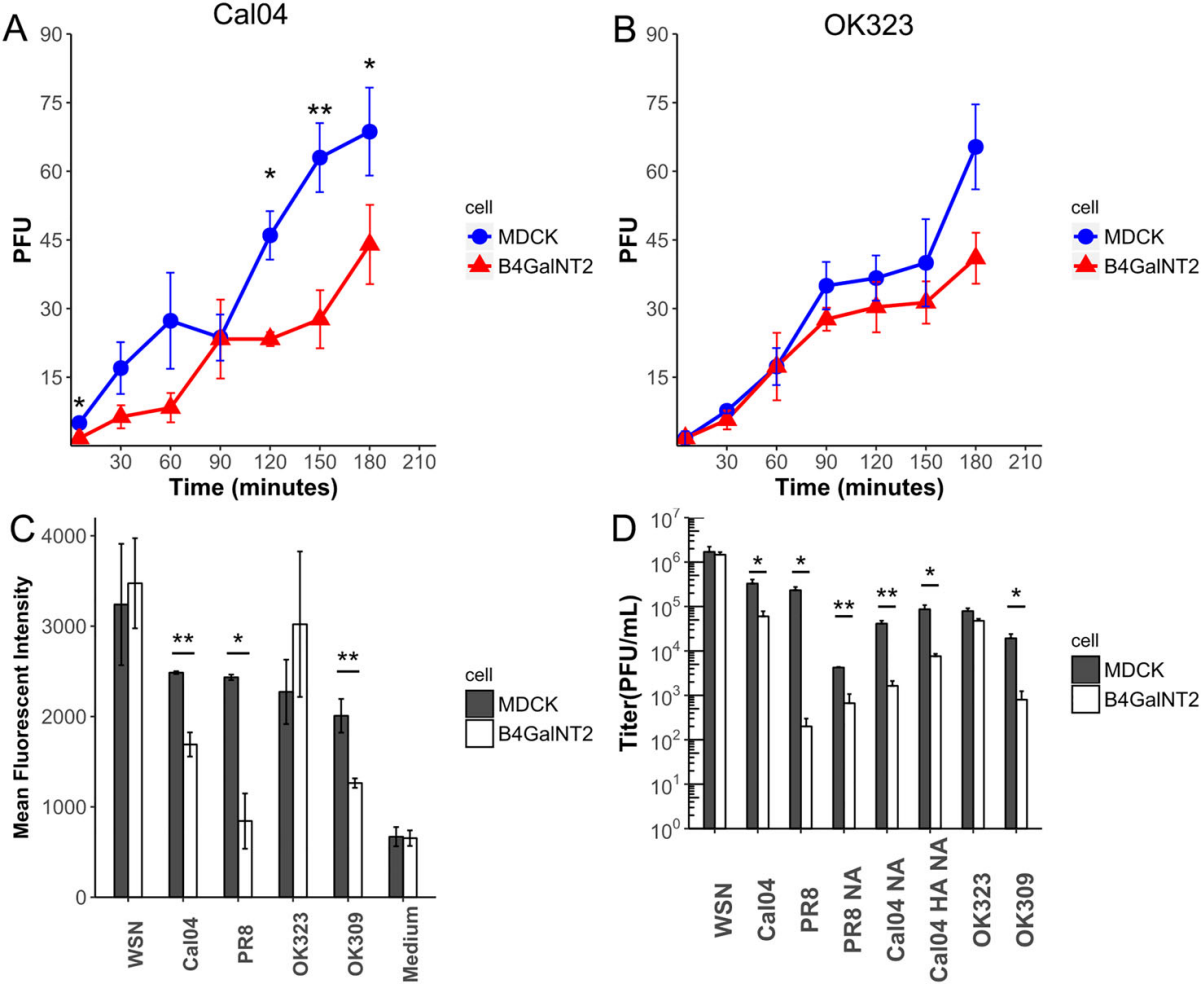
(A,B) Number of PFU formed after incubated with same amount of (A) Cal04 or (B) OK323 in plaque assay using MDCK and MDCK-B4GalNT2, which overlaid agarose at the time specified. (C) Proportion of NP positive cells normalized to that of wild type MDCK. Mean ± sd, *n* = 2. (D) MDCK and MDCK-B4GalNT2 were infected with influenza virus at MOI 1 for 8 h. Mean ± sd, *n* = 3. *p < 0.05, **p < 0.01, ***p < 0.001 by 2 tailed T-test.

To further confirm that inhibitions were not due to inhibition in establishment of viral infection, the rates of viral entry were compared. PFUs of the same dilution of virus were compared in plaque assays using MDCK and MDCK-B4GalNT2 cells overlaid with agarose at different time points (Figure 5(A,B)). PFUs of OK323 from both MDCK and MDCK-B4GalNT2 were coincided at all time points. For Cal04, despite plateau of similar levels of PFUs being observed, it was 30 min later in MDCK-B4GalNT2. The slope of PFU increase at the first 60 min was lower in MDCK-B4GalNT2. The results concluded that, despite same number of PFU eventually producing from the same viral spike, MDCK-B4GalNT2 required a longer incubation time for Cal04 infection.

### Viral inhibition by reduction of viral attachment

WSN, Cal04, OK323 and OK309 were labelled with R18 lipophilic fluorescent dye and allowed to attach to MDCK and MDCK-B4GalNT2 at 4°C (Figure 5(C)). The medium from uninfected MDCK was also labelled accordingly as a negative control. The mean fluorescent intensities (MFI) of the negative control were comparable, indicating background signal of both cells were comparable. In agreement with our previous experiments, significantly lower fluorescent levels were measured in Cal04, PR8 and OK309 attached MDCK-B4GalNT2, but that for WSN and OK323 were similar to MDCK. The results showed that attachments of Cal04, PR8 and OK309 were reduced in MDCK-B4GalNT2, but were comparable for WSN and OK323.

### HA and NA genes are important for the inhibition effects by MDCK-B4GalNT2

To show that viral HA and NA genes are the main determinants of the inhibition effect, WSN mutant strains, where HA and/or NA were substituted with Cal04 HANA, Cal04 NA and PR8 NA, were successfully produced by reverse genetics.

As shown in Figure 5(C), after infection at MOI 1 for 8 h, viral titres of Cal04, PR8 and OK309 were significantly decreased in MDCK-B4GalNT2 but were similar for WSN and OK323. Similar to wild type Cal04, with substitution of both HA and NA, Cal04 HANA resulted in a 10 fold lower titre in MDCK-B4GalNT2. Surprisingly, with only NA gene substituted, Cal04 NA and PR8 NA still showed 10 fold titre reductions in MDCK-B4GalNT2. The results indicated that NA of WSN is required for its efficient replication in MDCK-B4GalNT2.

### NA active sites are required for WSN NA aided entry

It has been reported that the α2,3-binding capability of OK309 was influenced by its NA active site that had reduced sialic acid cleavage activity due to the D151 mutation [6–8]. G147R mutation of N1, which can be found in a minor population in laboratory passaged virus, also enables active site-mediated receptor binding function with a slight decrease in NA activity [9,10]. Both NA mediated receptor binding mechanisms are sensitive to oseltamivir treatment [6–10].

**Figure 6.**
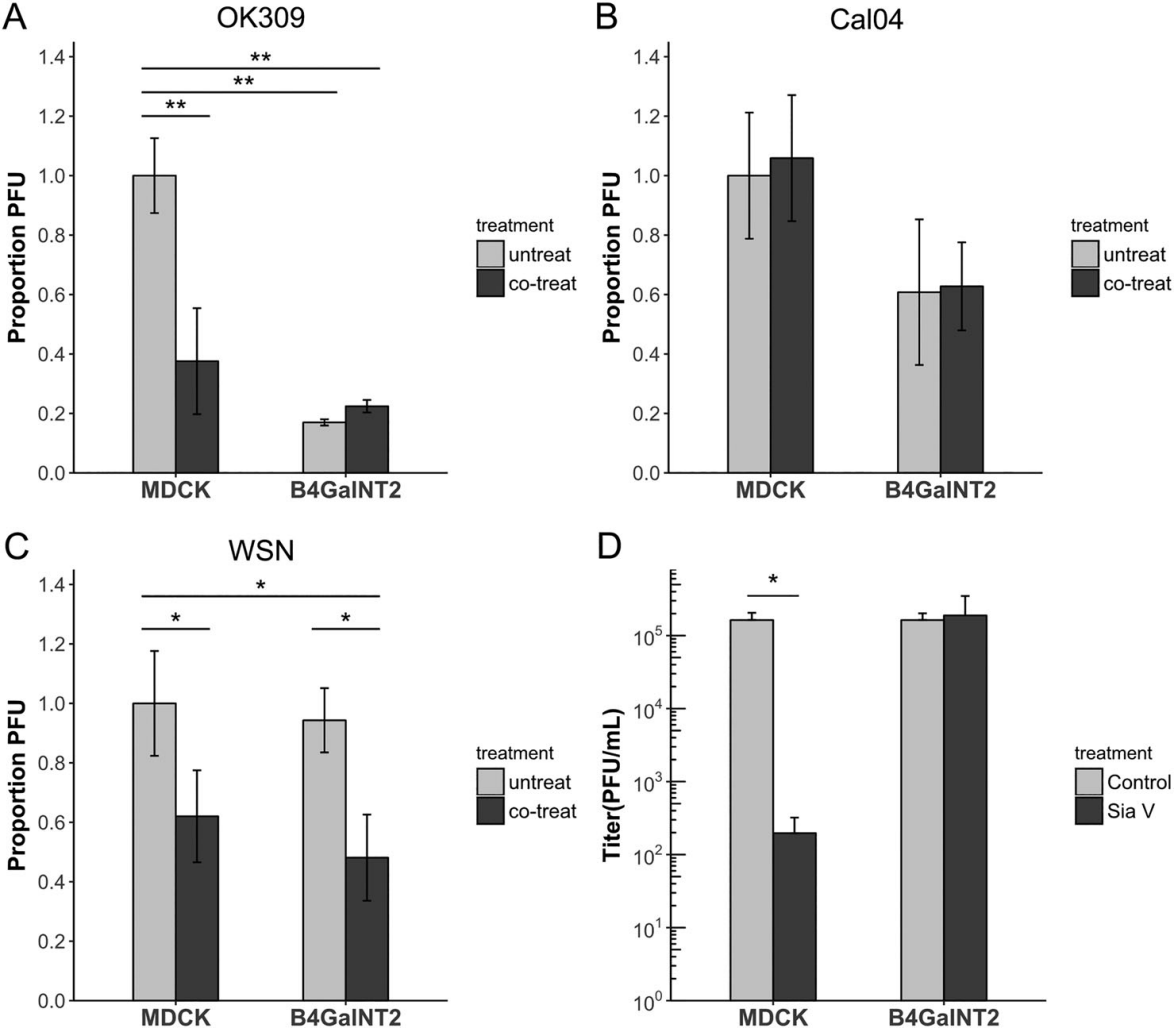
Plaque assay of MDCK and MDCK-B4GalNT2 infected with same amount of (A) OK309, (B) Cal04 or (C) WSN. 10nM oseltamivir carboxylate was added (co-treated) or not added (untreated) during viral entry. Results were normalized to untreated MDCK cells. (D) Sialidase V (SiaV) treatment of MDCK and MDCKB4GalNT2 prior to WSN infection at MOI 1 for 8 h. mean ± sd, *n* = 3. *p < 0.05, **p < 0.01, ***p < 0.001, 2 tailed T-test.

Plaque assays were performed on MDCK and MDCK-B4GalNT2 cells, without oseltamivir (untreated), with oseltamivir only during viral entry (co-treated) and with oseltamivir only after viral entry (post-treated). Plaques were so small to nearly invisible in post-treated wells for WSN, Cal04 and OK309 (data not shown), confirming that all three strains were sensitive to the applied concentration of oseltamivir. In concert with previous results that less virus can enter in a given time in MDCK-B4GalNT2, fewer plaques were formed in untreated wells for OK309 and Cal04 (Figure 6(A,B)), but similar PFU were present after WSN infection (Figure 6(C)). Co-treatment of oseltamivir significantly reduced the amount of plaque in OK309 infected MDCK but not in MDCK-B4GalNT2, suggesting that co-treating oseltamivir blocked OK309 NA active sites that are responsible for α2,3-binding and hence reduced viral entry (Figure 6(A)). Plaque reduction effect was absent in Cal04 co-treated samples, indicating NA active sites of Cal04 are not responsible for its α2,3-binding (Figure 6(B)). On the contrary, less plaques were formed in both MDCK and MDCK-B4GalNT2 co-treated WSN samples than that in untreated samples, indicating WSN NA may serve to bind non-α2,3-receptors to aid viral entry (Figure 6(C)).

### WSN infection requires sialic acids

Finally to test the possibility that WSN utilizes non-sialic acid receptors for entry, MDCK and MDCK-B4GlNT2 were treated with a broad spectrum sialidase, Vibrio Sialidase (SiaV), to remove available receptors before infection. Viral titres of control and SiaV treated MDCK-B4GalNT2 were comparable, suggesting strong sialidase resistance of Sda epitopes (Figure 6(D)). On the other hand, viral titre in MDCK dropped significantly upon SiaV treatment, which indicates WSN indeed requires sialic acids for entry (Figure 6(D)).

## Discussion

We have shown that MDCK-B4GalNT2 cells inhibited attachments and infections by the majority of α2,3-sialic acid binding influenza viruses with the exception of WSN. Infection by two 2009 pandemic H1N1 strains, Cal04 and HK09, previously shown to bound only to α2,6-sialic acids by glycan array, was also inhibited in MDCK-B4GalNT2. Inhibition of viral infection was due to reduced viral entry rate and attachment.

Whole virus and HA of Cal04 were reported to bind only α2,6-receptors according to multiple glycan arrays from CFG (primscreen_2777, 3103, 3104, 3128) and previous publications [36–39]. However, computational modelling and glycolipid-based microarray have shown that Cal04 may bind to both α2,3- and α2,6-linked receptors [40,41]. Although crystal structure analysis showed Cal04 HA binds more stably to human-like receptors, conserved interactions around the terminal sialic acid were found for both α2,3- and α2,6-receptors [42]. Recent publications using NMR spectroscopy have also shown that Cal04, previously considered a α2,6 binding virus, may bind to α2,3 terminated O = glycans [43]. Indeed the current experiments indicated that the 2009 pandemic H1N1 strains, Cal04 and HK09 require α2,3-linked receptors for efficient viral infection, and the α2,3-receptor binding capability of Cal04 was not mediated by its NA active site.

On the contrary, WSN behaves like an α2,6-binding only virus. Despite WSN showed only α2,3-binding capability in the majority of glycan arrays in online data base (CFG primscreen_2664, 2933, 3720, 5336), it was also claimed to bind α2,6-receptors in some reports [11,34,35]. One report showed that WSN binds to most α2,3-receptors tested, but can only bind to α2,6-receptors on antennary N-glycans with multiple N-acetyllactosamine repeats, but not short synthetic receptors[11]. HA and NA balance is believed to be vital to viral fitness. NA of some influenza strains have evolved to assist in receptor binding [6–10]. By replacing NA of WSN with that from PR8 or Cal04, we showed that WSN became sensitive to the depletion of α2,3-receptors. This may be due to the requirement of NA of WSN for initial binding of virus, despite remaining capable of receptor cleavage [11].

DK/Bav binds α2,3-binding only, but limited viral replication was still observed in MDCK-B4GalNT2. B4GalNT2 although can efficiently modify sialic acid receptors on N-glycans and O-glycans on glycoproteins, some glycolipids, including GM3, are modified only by β-1,4-N-Acetyl-Galactosaminyltransferase 1 (B4GalNT1) [16]. Very weak bindings to α2,6-receptors were shown in glycan array data for DK/Bav [4]. Hence, DK/Bav may still able to bind to the unmodified α2,3-receptors on glycolipids or weakly to α2,6-receptors to infect MDCK-B4GalNT2.

Mass spectrometric analysis of tissues from the human respiratory tract has shown that there is a high relative abundance of biantennary complex glycans with α2,3 receptor on one branch of the antenna and α2,6 on the other [4]. Glycan array has shown that Cal04 and WSN bind to these biantennary α2,3/α2,6 glycans. In the case of MDCK-B4GalNT2 the α2,3 arm is modified to Sda, and even though there is reduced binding to this antenna, the other α2,6 allows WSN binding and infection. Recent publications using NMR spectroscopy have also shown that Cal04, previously considered a α2,6 binding virus, may bind to α2,3 terminated O-glycans [43]. This raises the possibility that the flexible binding preference of some influenza viruses to the glycan may be a positive factor determining their success at replication.

Though glycan array is useful as a mass screening platform, the use of MDCK-B4GalNT2 should be considered as an additional tool, as it resembles most aspects of a normal cell and can be performed in physiological conditions. O-glycans and N-glycans are 3-dimensionally presented on native protein instead of a flat solid support. Various glycans are displayed together on the cell surface at physiological density. Membrane fluidity, glycolipids and micro-domains are conserved. Both viral attachment and internalization can be studied. The effect of B4GalNT2 gene is more specific, effective and consistent than bacterial sialidases that remove only α2,3-sialic receptors, as α2,6-receptors are not the substrates of B4GalNT2. By just comparing PFUs in plaque assay using MDCK and MDCK-B4GalNT2, which basically correlates to viral entry and single cycle growth kinetic, α2,3-sialic receptors requirement of an influenza strain can be easily determined.
